# Complete Tumor Resection and Radical Lymphadenectomy: Potential Cure for Adrenocorticotropic Hormone (ACTH)-Dependent Pulmonary Carcinoid

**DOI:** 10.7759/cureus.73438

**Published:** 2024-11-11

**Authors:** Arwa Elsheikh, Inga Harbuz-Miller, Edward Vates, Michael Nead, Ismat Shafiq

**Affiliations:** 1 Endocrinology, Diabetes, and Metabolism, UCHealth Anschutz Outpatient Pavilion, Anschutz Medical Campus, Aurora, USA; 2 Endocrinology and Metabolism, University of Rochester School of Medicine and Dentistry, Rochester, USA; 3 Neurosurgery, University of Rochester School of Medicine and Dentistry, Rochester, USA; 4 Pulmonary and Critical Care Medicine, University of Rochester School of Medicine and Dentistry, Rochester, USA

**Keywords:** acth-dependent cushing syndrome, hypercortisolemia, lung surgery, lymph node dissection, pulmonary carcinoids

## Abstract

Ectopic adrenocorticotropic hormone (ACTH)-dependent Cushing is a rare syndrome. We present a case that illustrates the diagnostic and therapeutic challenges of ectopic Cushing. A 35-year-old woman presented to the outpatient clinic for evaluation of progressive weight gain, muscle weakness, easy bruising, uncontrolled hypertension, and hyperglycemia. Biochemical workup revealed elevated salivary cortisol and 24-hour urine cortisol; the baseline ACTH was elevated, consistent with ACTH-dependent hypercortisolemia. Imaging showed a pituitary microadenoma and a lung nodule. Inferior petrosal sinus sampling was suggestive of an ectopic source. Medical treatment was employed to manage acute hypercortisolemia with a resolution of symptoms. A biopsy of the lung nodule showed the neuroendocrine tumor. Surgical treatment with pulmonary wedge resection did not alleviate hypercortisolemia, leading to repeat surgery with radical lymph node dissection, which resulted in the resolution of hypercortisolemia. This case illustrates that radical lymph node dissection, along with tumor resection, has a high likelihood of cure.

## Introduction

Ectopic adrenocorticotropic hormone (ACTH)-dependent Cushing syndrome (CS) is rare. The incidence is about 0.7-2.4 cases per million [1]. Ectopic CS accounts for about 8 to 20% of ACTH-dependent CS [1], with 50% found in the lungs [2,3]. Ectopic ACTH-secreting pulmonary carcinoid (EAPC) represents 25% of lung lesions. Medical or surgical approaches are applied to treat hypercortisolemia while figuring out the source [4]. Surgical removal of the tumor is the mainstay treatment for EAPC; however, the extent of pulmonary surgery can impact the cure [5]. Our case identifies the challenges associated with the surgical treatment of EAPC.

## Case presentation

A 35-year-old woman presented to the outpatient clinic with weight gain of over 50 pounds in one year, progressive muscle weakness, new hirsutism, acne, easy bruising, worsening depression, anxiety, and new-onset hypertension and hyperglycemia. Her past medical history included a stable lung nodule diagnosed eight years ago and a Roux-en-Y gastric bypass and ankle fracture after tripping over one step. Initial evaluation revealed elevated blood pressure at 162/87 mmHg, with a weight of 214 lbs, and a body mass index of 41.6. She appeared cushingoid with a round, plethoric face, excessive facial hair, acne, supraclavicular fat pad, and wide purple striae over the abdomen. The laboratory evaluation was consistent with ACTH-dependent CS, showing morning cortisol levels of 35.1 ug/dl (6-18.4 ug/dl), ACTH levels of 157 pg/ml (7-53 pg/ml), 24-hour urine-free cortisol levels of 610 mcg/24 hour (4-50 mcg/24 hours), and two midnight salivary cortisol levels of 1420 and 1470 ng/dl (0-99 ng/dl) (Table 1).

**Table 1:** Summary of the biochemical testing for evaluation of ACTH-dependent hypercortisolemia. ACTH: adrenocorticotropic hormone; MN: midnight; UFC: urinary free cortisol; HDDST: high-dose dexamethasone suppression test

Test	Result	Reference range with unit
ACTH	157 pg/ml	7.0-63 pg/ml
Cortisol	35.1 ug/dl	6.0-18.4ug/dl
MN salivary cortisol	1420 and 1470 ng/dl	0-99 ng/dl
24-hour UFC	610.1 mcg/24hr	4.0-50.0 mcg/24hr
HDDST	30.2ug/dl	

**Figure 1:**
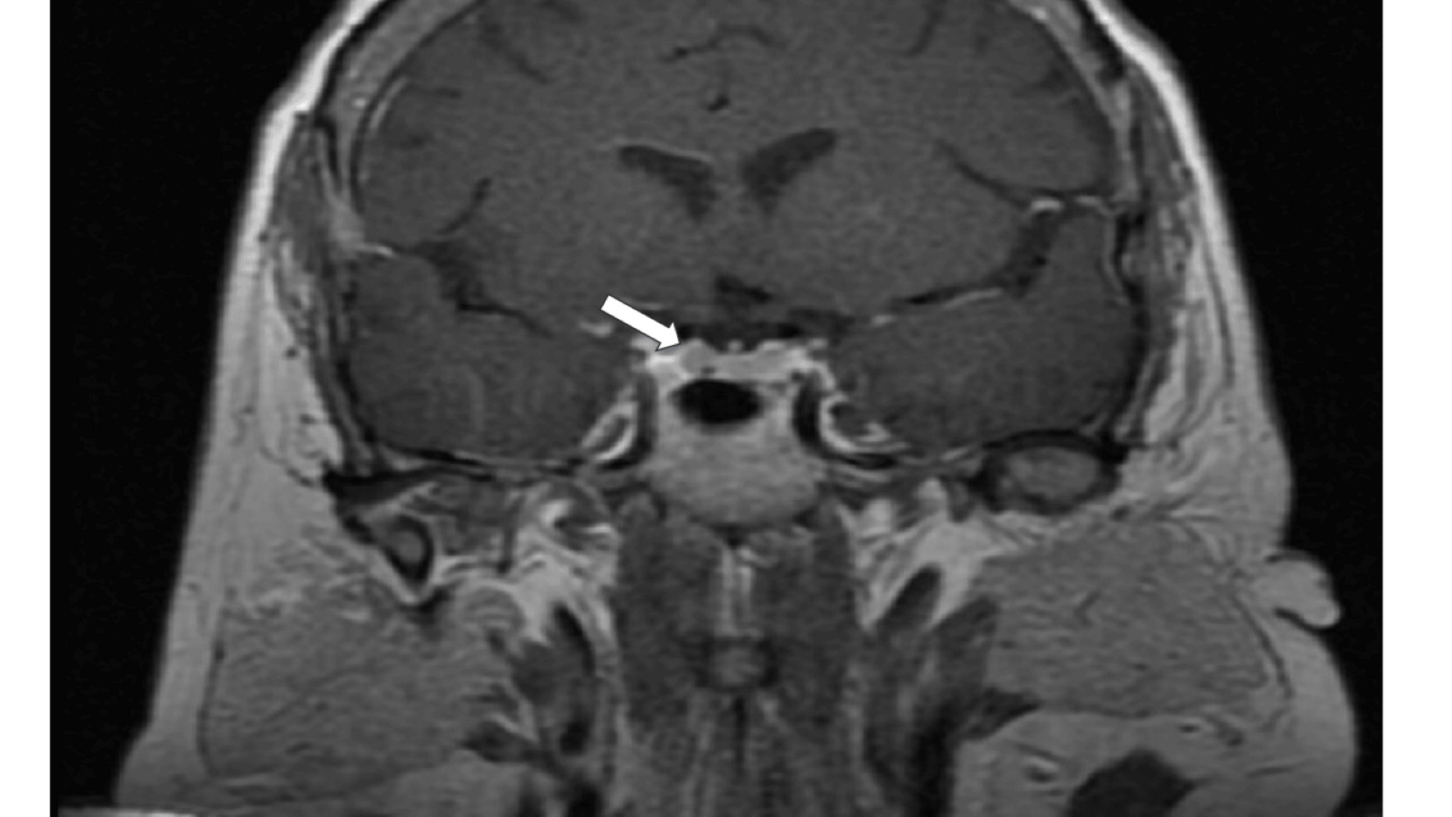
Magnetic resonance imaging (MRI) head with a 4 mm pituitary microadenoma (white arrow).

**Figure 2:**
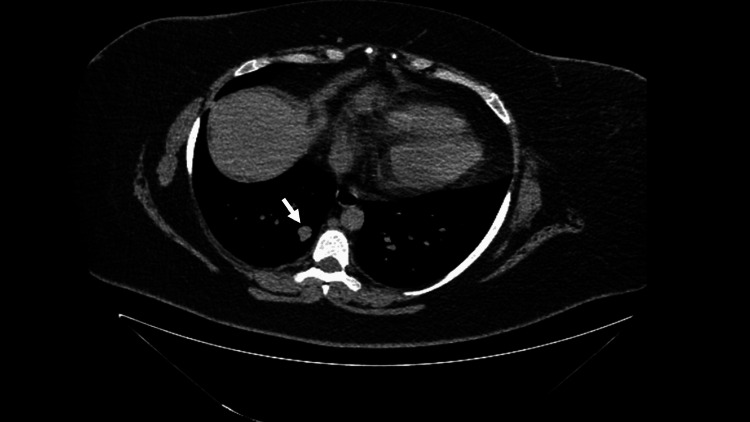
Computed tomography (CT) of the chest showed a right 1.2 cm lung nodule (white arrow).

A dedicated pituitary magnetic resonance imaging (MRI) revealed a 4 mm pituitary microadenoma (Figure 1). The chest computed tomography (CT) indicated that the lung nodule measuring 1.2 cm (Figure 2) has remained stable over eight years.

**Figure 3:**
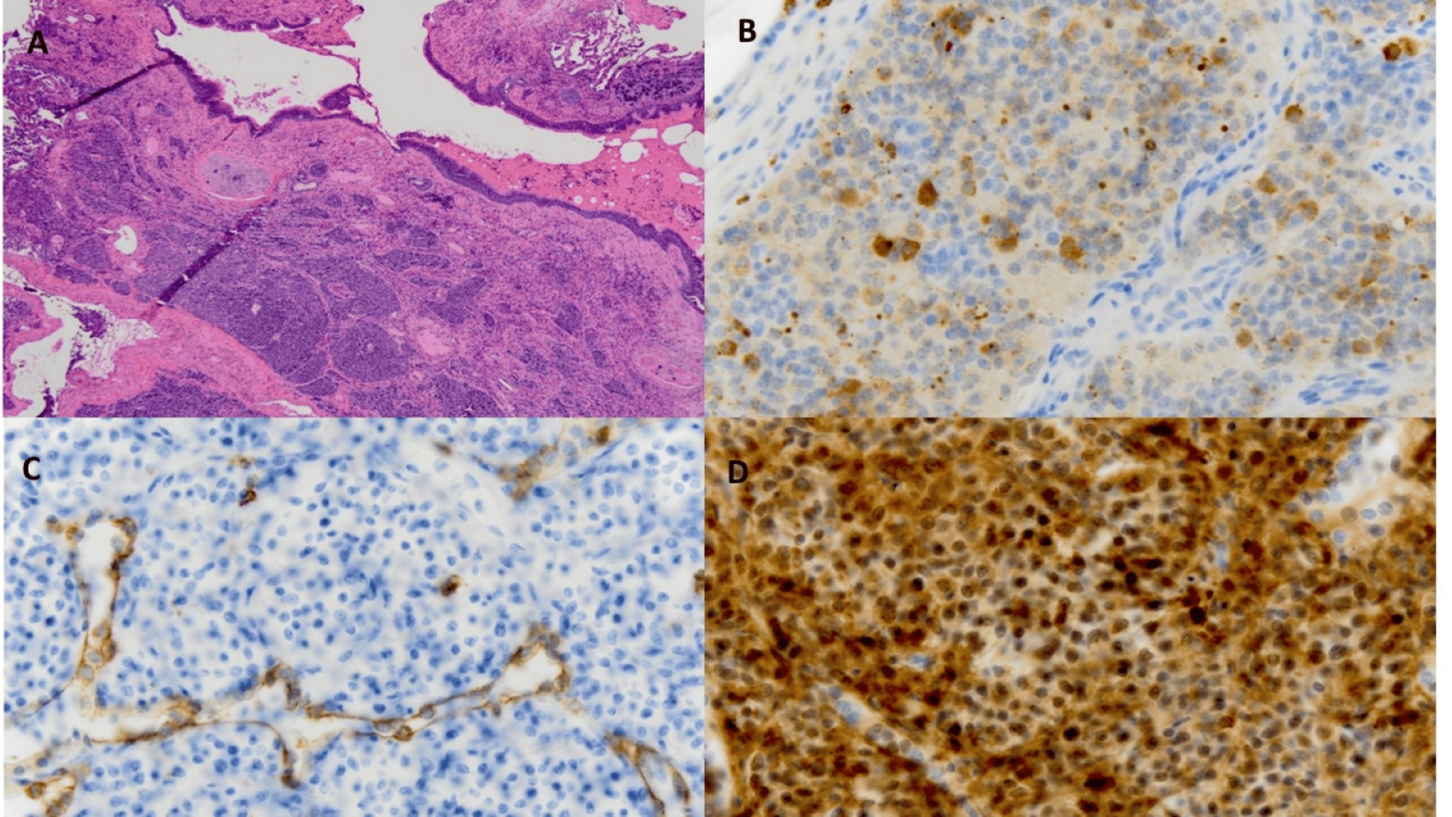
Histopathology of the right lower lung nodule showed a carcinoid tumor. A: hematoxylin and eosin staining, B: ACTH expression, C: pan-cytokeratin staining, D: chromogranin staining ACTH: adrenocorticotropic hormone

Given her complex history, pituitary microadenoma, and history of a lung nodule, bilateral inferior petrosal sinus sampling (BIPSS) was performed, which suggested an ectopic CS. Functional imaging, including the octreotide scan, fluorodeoxyglucose (FDG)-positron emission tomography (PET)/magnetic resonance (FGDPET/MR), and gallium-68-DOTATATE PET/CT (Ga-68 PET/CT) scan, was unrevealing. A CT-guided biopsy of the lung nodule revealed a neuroendocrine tumor, a typical carcinoid. The histopathology specimen was positive for pan-cytokeratin, chromogranin (focal), and ACTH (focal) with Ki67 < 1% (Figure 3).

With severe hypercortisolemia, the patient suffered fragility fractures with vertebral compression and recurrent urinary tract infections, leading to hospitalization with acute renal failure. Medical treatment with steroidogenesis inhibitors was initiated with metyrapone and ketoconazole, which were titrated to achieve normal cortisol levels. Following stabilization, the patient underwent right lower lobe pulmonary wedge resection, and histopathology confirmed a typical carcinoid tumor. Postoperatively, the patient remains hypercortisolemic, and metyrapone was resumed and titrated to achieve an eucortisolemic state. Six months post-op, the patient underwent right lower lobe completion lobectomy and lymph node dissection, leading to the normalization of cortisol of medical treatment. Surgical pathology was positive for a 0.6 cm residual parenchymal and perivascular carcinoid tumor. Twelve loco-regional lymph nodes were dissected and negative for malignancy. Postoperatively, the patient developed adrenal insufficiency, requiring glucocorticoid replacement. The glucocorticoid regimen was titrated over 18 months and discontinued after the hypothalamo-pituitary-adrenal axis function returned to normal. The patient remained normotensive and normoglycemic post-op. She remained in remission six years after the curative surgery.

## Discussion

**Figure 4:**
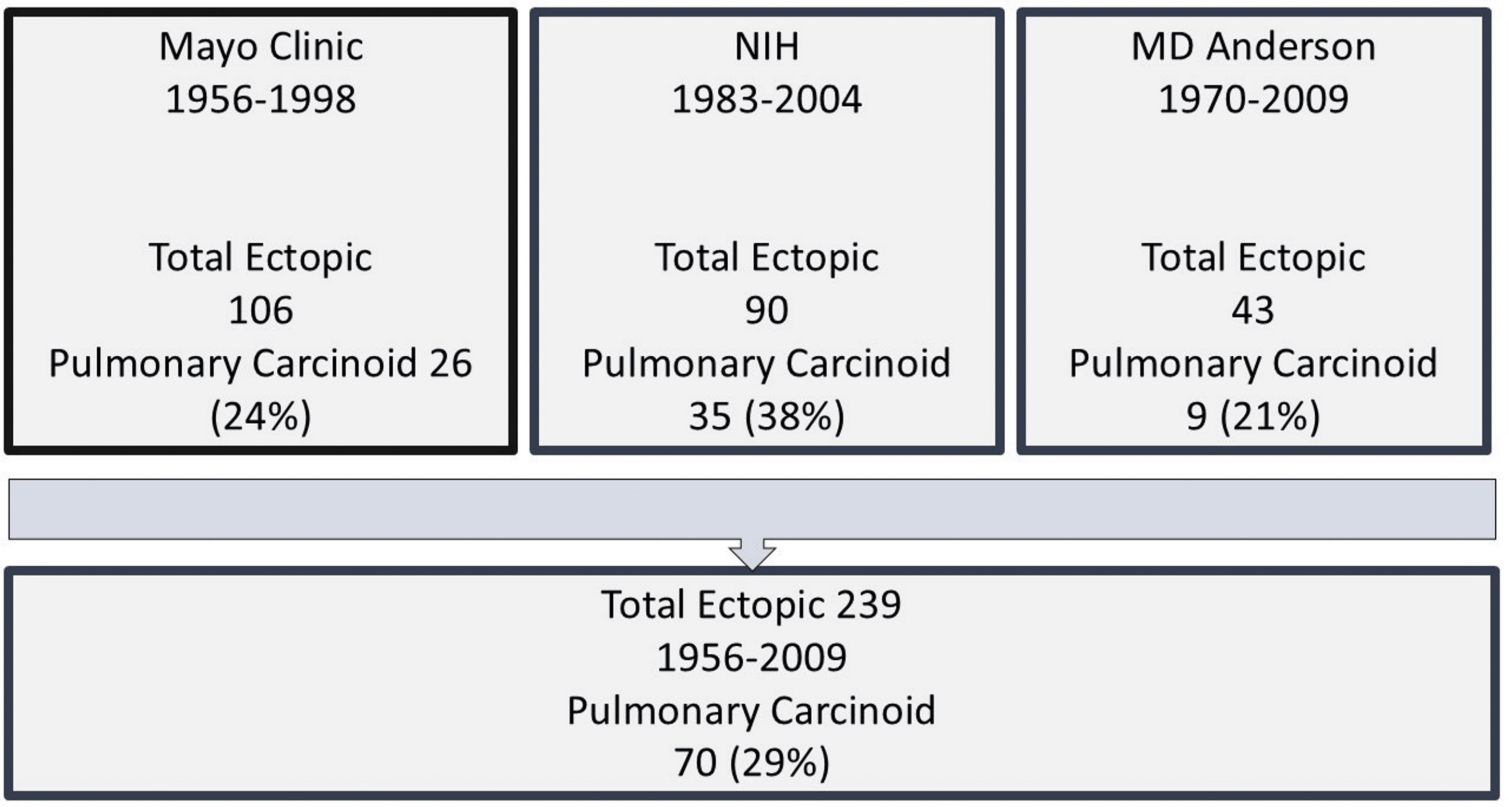
Total cases of pulmonary carcinoid reported in large series in the United States. [3,6,7]

Ectopic ACTH-secreting tumors causing hypercortisolemia are rare. Meador and Little et al. described a series of five patients with ACTH-secreting lung tumors in 1968, elucidating their role in hypercortisolemia [2]. While ACTH is typically produced by neuroendocrine tumors located in the thymus, pancreas, thyroid, and adrenal glands, pulmonary tumors account for approximately 50% of cases, with half being carcinoid [1]. The exact incidence of ectopic ACTH-secreting pulmonary carcinoids (EAPC) is challenging to extrapolate from the case reports. Between 1956 and 2009, 239 ectopic Cushing cases were reported in the United States (Figure 4) [3,6,7]. Among the 239 cases, 70 cases were EAPC, constituting 29% of total ectopic Cushing [3,6,7]. A UK-based study reported twelve patients with EAPC, representing 30% of all ectopic Cushing [4]. A recent study from Southern India showed 33% bronchial carcinoid in all ectopic Cushing over ten years [8].

Tumor localization can take months to years, ranging between one and 264 months [5,9,10]. Conventional imaging with CT and MRI of the chest can identify ectopic ACTH sources in up to 70-90% of the cases [1]. Shrager et al. suggested that a chest CT scan every three to six months might reveal the source [10]. Functional imaging with octreotide, FDG-PET/CT, or PET/MR, 18 F-DOPA L-6-[18F] fluoro-3,4-dihydroxyphenylalanine (18F-DOPA) positron emission tomography (PET) (18 F-DOPA PET), and Ga-68 PET/CT scans can detect the pulmonary carcinoid tumor, all with different sensitivities ranging from 14 to 94% [11]. In our cases, we had a known diagnosis of stable pulmonary nodule. The FDG-PET nor the Ga-68 PET/CT revealed the source in our case, leading to a biopsy of the stable right lung nodule.

The mortality and morbidity associated with EAPC are related to cortisol excess and its comorbidities [9]. Eucortisolemia can be achieved with bilateral adrenalectomy or with medical treatment when the primary source is unclear. Bilateral adrenalectomy is appropriate in patients with failed medical therapy, and occult, or unresectable tumors. Although adrenalectomy rapidly normalizes cortisol, it may not be an ideal long-term treatment option in EPAC. Several medical therapy options are available to treat hypercortisolemia [9,12,13]. Steroidogenesis inhibitors (metyrapone, ketoconazole, and osilodrostat) are the mainstay of medical treatment. Our patient needed steroidogenesis inhibitors to normalize cortisol levels. A glucocorticoid receptor antagonist is used in a small case series. Other treatment options are somatostatin analogs and dopamine agonists with limited experience [14].

The frequent involvement of lymph nodes makes the EAPC an aggressive variant [5,9,11,15,16]. Due to their advanced nature, most authors recommend a complete EAPC resection and radical lymphadenectomy treating them as high-grade malignant tumors [5,9-11,15,17]. There are several isolated case reports of EAPC, but only a handful of case series with details on surgery and postoperative outcomes, as summarized in Table 2. Pass et al. published the initial series of fourteen patients, emphasizing the extent of surgery with lymph node dissection to increase the chances of cure [5]. Boddart et al. further reported no recurrence over a mean follow-up of 57 months after extensive surgery [9]. A recent study by Lococo et al. showed a lower risk of recurrence with tumor resection and lymphadenectomy [11]. Shrager et al. had seven patients with EACP. Three had only wedge resection, leading to repeat surgery with lymph node dissection. The recurrence rate is 0-23% with tumor and regional lymph node dissection but up to 57% with local resection only [10]. Several case reports showed better outcomes with surgical resection and regional lymphadenectomy [18-20]. It’s not clear from the case series how often the LN were involved, but the cure rate has been higher in patients with lobectomy and LN dissection.

**Table 2:** Data from large case series on the surgical outcome of EAPC. EAPC: ectopic adrenocorticotropic secreting pulmonary carcinoid; LN: lymph node; N: number

Author	Total number of patients with pulmonary carcinoid	Lung resection	LN dissection	Recurrence/persistent hypercortisolemia after 1^st^ surgery N (%)
Pass et al., 1990 [5]	13	13/13	12/13	3(23)
Zeiger et al., 1992 [17]	20	20/20	20/20	2(10)
Shrager et al., 1997 [10]	7	7/7	4/7	4(57)
Deb et al., 2005 [15]	22	22	19	4(18)
Boddaert et al., 2012 [9]	14	14	14	0
Lococo et al., 2016 [11]	23	21	21	5(23)
La Rosa et al., 2019 [16]	11	11	11	2(18)
Total	110	110	104	22(20%)

## Conclusions

ACTH-secreting pulmonary carcinoid tumors are a rare cause of ectopic Cushing syndrome. Their diagnosis remains difficult and often delayed despite the use of multiple advanced diagnostic techniques. Medical treatment can achieve eucortisolemia while identifying the source. Due to the aggressive nature of these tumors, with a high prevalence of lymph node involvement, the surgical treatment should aim for complete anatomic resection and radical lymphadenectomy as it increases the cure rate.

## References

[1] Meador CK, Liddle GW, Luetscher JA (1962). Cause of Cushing's syndrome in patients with tumors arising from "nonendocrine" tissue. J Clin Endocrinol Metab.

[2] Hayes AR, Grossman AB (2018). The ectopic adrenocorticotropic hormone syndrome: rarely easy, always challenging. Endocrinol Metab Clin North Am.

[3] Ejaz S, Vassilopoulou-Sellin R, Busaidy NL (2011). Cushing syndrome secondary to ectopic adrenocorticotropic hormone secretion: the University of Texas MD Anderson Cancer Center Experience. Cancer.

[4] Isidori AM, Kaltsas GA, Pozza C (2006). The ectopic adrenocorticotropin syndrome: clinical features, diagnosis, management, and long-term follow-up. J Clin Endocrinol Metab.

[5] Pass HI, Oldfield EH, Cutler GB (1990). Management of the ectopic ACTH syndrome due to thoracic carcinoids. Ann Thorac Surg.

[6] Aniszewski JP, Young WF Jr, Thompson GB, Grant CS, van Heerden JA (2001). Cushing syndrome due to ectopic adrenocorticotropic hormone secretion. World J Surg.

[7] Ilias I, Torpy DJ, Pacak K, Mullen N, Wesley RA, Nieman LK (2005). Cushing's syndrome due to ectopic corticotropin secretion: twenty years' experience at the National Institutes of Health. J Clin Endocrinol Metab.

[8] Sathyakumar S, Paul TV, Asha HS (2017). Ectopic Cushing syndrome: A 10-year experience from a tertiary care center in southern India. Endocr Pract.

[9] Boddaert G, Grand B, Le Pimpec-Barthes F, Cazes A, Bertagna X, Riquet M (2012). Bronchial carcinoid tumors causing Cushing's syndrome: more aggressive behavior and the need for early diagnosis. Ann Thorac Surg.

[10] Shrager JB, Grillo HC, Mathisen DJ (1997). Bronchopulmonary carcinoid tumors associated with Cushing's syndrome: a more aggressive variant of typical carcinoid. J Thorac Cardiovasc Surg.

[11] Lococo F, Margaritora S, Cardillo G (2016). Bronchopulmonary carcinoids causing Cushing syndrome: results from a multicentric study suggesting a more aggressive behavior. Thorac Cardiovasc Surg.

[12] Feelders RA, Hofland LJ, Lacroix A, Newell-Price J, Pivonello R, Nieman LK (2019). Advances in the medical treatment of Cushing's syndrome. Lancet Diabetes Endocrinol.

[13] Pivonello R, Ferrigno R, De Martino MC (2020). Medical treatment of Cushing’s disease: an overview of the current and recent clinical trials. Front Endocrinol (Lausanne).

[14] de Bruin C, Feelders RA, Lamberts SW, Hofland LJ (2009). Somatostatin and dopamine receptors as targets for medical treatment of Cushing's syndrome. Rev Endocr Metab Disord.

[15] Deb SJ, Nichols FC, Allen MS, Deschamps C, Cassivi SD, Pairolero PC (2005). Pulmonary carcinoid tumors with Cushing's syndrome: an aggressive variant or not?. Ann Thorac Surg.

[16] La Rosa S, Volante M, Uccella S (2019). ACTH-producing tumorlets and carcinoids of the lung: clinico-pathologic study of 63 cases and review of the literature. Virchows Arch.

[17] Zeiger MA, Pass HI, Droppman JD (1992). Surgical strategy in the management of non-small cell ectopic adrenocorticotropic hormone syndrome. Surgery.

[18] Sakuraba M, Murasugi M, Oyama K, Adachi T, Ikeda T, Onuki T (2003). Diagnosis and surgical treatment of ectopic adrenocorticotropic hormone-producing pulmonary tumors accompanied by Cushing syndrome. Jpn J Thorac Cardiovasc Surg.

[19] Savu C, Melinte A, Lukadi JL (2020). Neuroendocrine syndrome in bronchial carcinoid tumors. Exp Ther Med.

[20] de Matos LL, Trufelli DC, das Neves-Pereira JC, Danel C, Riquet M (2006). Cushing's syndrome secondary to bronchopulmonary carcinoid tumor: report of two cases and literature review. Lung Cancer.

